# Differentiation in stem and leaf traits among sympatric lianas, scandent shrubs and trees in a subalpine cold temperate forest

**DOI:** 10.1093/treephys/tpab049

**Published:** 2021-04-05

**Authors:** Ke-Yan Zhang, Da Yang, Yun-Bing Zhang, David S Ellsworth, Kun Xu, Yi-Ping Zhang, Ya-Jun Chen, Fangliang He, Jiao-Lin Zhang

**Affiliations:** CAS Key Laboratory of Tropical Forest Ecology, Xishuangbanna Tropical Botanical Garden, Chinese Academy of Sciences, Menglun, Mengla 666303, Yunnan, China; Center of Plant Ecology, Core Botanical Gardens, Chinese Academy of Sciences, Menglun, Mengla 666303, Yunnan, China; University of Chinese Academy of Sciences, Yuquan Road 19A, Beijing 100049, China; CAS Key Laboratory of Tropical Forest Ecology, Xishuangbanna Tropical Botanical Garden, Chinese Academy of Sciences, Menglun, Mengla 666303, Yunnan, China; Center of Plant Ecology, Core Botanical Gardens, Chinese Academy of Sciences, Menglun, Mengla 666303, Yunnan, China; CAS Key Laboratory of Tropical Forest Ecology, Xishuangbanna Tropical Botanical Garden, Chinese Academy of Sciences, Menglun, Mengla 666303, Yunnan, China; Center of Plant Ecology, Core Botanical Gardens, Chinese Academy of Sciences, Menglun, Mengla 666303, Yunnan, China; University of Chinese Academy of Sciences, Yuquan Road 19A, Beijing 100049, China; Hawkesbury Institute for the Environment, Western Sydney University, Locked Bag 1797, Penrith NSW 2751, Australia; Lijiang Forest Ecosystem Research Station, Kunming Institute of Botany, Chinese Academy of Sciences, Lijiang 674100, Yunnan, China; CAS Key Laboratory of Tropical Forest Ecology, Xishuangbanna Tropical Botanical Garden, Chinese Academy of Sciences, Menglun, Mengla 666303, Yunnan, China; Center of Plant Ecology, Core Botanical Gardens, Chinese Academy of Sciences, Menglun, Mengla 666303, Yunnan, China; CAS Key Laboratory of Tropical Forest Ecology, Xishuangbanna Tropical Botanical Garden, Chinese Academy of Sciences, Menglun, Mengla 666303, Yunnan, China; Center of Plant Ecology, Core Botanical Gardens, Chinese Academy of Sciences, Menglun, Mengla 666303, Yunnan, China; Department of Renewable Resources, University of Alberta, Edmonton, Alberta T6G 2H1, Canada; CAS Key Laboratory of Tropical Forest Ecology, Xishuangbanna Tropical Botanical Garden, Chinese Academy of Sciences, Menglun, Mengla 666303, Yunnan, China; Center of Plant Ecology, Core Botanical Gardens, Chinese Academy of Sciences, Menglun, Mengla 666303, Yunnan, China

**Keywords:** ecological strategy, functional traits, growth form, high elevation, leaf habit, nutrient stoichiometry, woody vine

## Abstract

The scandent shrub plant form is a variant of liana that has upright and self-supporting stems when young but later becomes a climber. We aimed to explore the associations of stem and leaf traits among sympatric lianas, scandent shrubs and trees, and the effects of growth form and leaf habit on variation in stem or leaf traits. We measured 16 functional traits related to stem xylem anatomy, leaf morphology and nutrient stoichiometry in eight liana, eight scandent shrub and 21 tree species co-occurring in a subalpine cold temperate forest at an elevation of 2600–3200 m in Southwest China. Overall, lianas, scandent shrubs and trees were ordered along a fast–slow continuum of stem and leaf functional traits, with some traits overlapping. We found a consistent pattern of lianas > scandent shrubs > trees for hydraulically weighted vessel diameter, maximum vessel diameter and theoretical hydraulic conductivity. Vessel density and sapwood density showed a pattern of lianas = scandent shrubs < trees, and lianas < scandent shrubs = trees, respectively. Lianas had significantly higher specific leaf area and lower carbon concentration than co-occurring trees, with scandent shrubs showing intermediate values that overlapped with lianas and trees. The differentiation among lianas, scandent shrubs and trees was mainly explained by variation in stem traits. Additionally, deciduous lianas were positioned at the fast end of the trait spectrum, and evergreen trees at the slow end of the spectrum. Our results showed for the first time clear differentiation in stem and leaf traits among sympatric liana, scandent shrub and tree species in a subalpine cold temperate forest. This work will contribute to understanding the mechanisms responsible for variation in ecological strategies of different growth forms of woody plants.

## Introduction

Forests support several coexisting woody growth forms, of which the most common are trees, multi-stemmed shrubs and lianas (Schnitzer 2018). Lianas (woody vines) are structural parasites that cannot stand erectly by themselves and require mechanical support from other plants to complete their life cycles (Darwin 1865, Jiménez-Castillo and Lusk 2013, Schnitzer 2018). Lianas usually have narrow stems and allocate more resources for leaf growth and/or stem elongation (Wyka et al. 2013, Ichihashi and Tateno 2015, Ganthaler et al. 2019), though a recent study found that lianas allocate proportionally similar biomass to stems as trees (Smith-Martin et al. 2020). To support a larger leaf area per cross-sectional stem area, lianas have evolved wide and long vessels, which transport water and nutrients fast and efficiently (Wyka et al. 2013, Chen et al. 2015, Ganthaler et al. 2019). Therefore, in contrast to trees, most studies suggest that lianas are aligned towards the fast-growth/resource acquisition end of the global trait spectra (Wright et al. 2004, Wyka et al. 2013, 2019, Werden et al. 2017).

Some studies have pointed out that lianas have two different forms in their sapling stage: climbing and self-supporting (Rowe and Speck 2005, van der Sande et al. 2013, Chen et al. 2014, Campanello et al. 2016). The self-supporting form has upright and self-supporting stems when young, but in older stages of development becomes climbing on a host or begins to bend without a host. This form has been called ‘scandent shrub’ to distinguish from ‘true’ lianas (Lahaye et al. 2005, Campanello et al. 2016). Stem anatomy and biomechanics could reflect an important structural differentiation between scandent shrubs and lianas (Isnard and Silk 2009, Chen et al. 2014, Campanello et al. 2016). For example, Chen et al. (2014) found that a scandent shrub has higher stem rigidity as indicated by Young’s modulus but lower specific stem hydraulic conductivity than a liana at their mature stages. Nevertheless, there are still few studies that have compared stem traits between lianas and scandent shrubs and questions remain about the degree of structural differentiation between these two growth forms.

Differentiation in functional traits among lianas, scandent shrubs and trees may be particularly associated with stem traits, considering that lianas are unable to stand upright, scandent shrubs go through two different stages from self-supporting to climbing, and trees are always upright. Previous studies have shown that lianas have larger vessels, higher hydraulic conductivity and lower wood density than co-occurring trees (Zhu and Cao 2009, Jiménez-Castillo and Lusk 2013, Campanello et al. 2016). However, some studies have also shown that the vessel diameter of lianas is smaller than that of co-occurring trees (Werden et al. 2017) and no differences have been found in their xylem-specific hydraulic conductivity and wood density (van der Sande et al. 2013, Zhang et al. 2019). Campanello et al. (2016) pointed out that liana seedlings should invest more resources for rapid elongation and a more efficient water transport system than scandent shrub seedlings. These studies have included just a few scandent shrub species, so how these three coexisting growth forms differ in their stem traits needs further investigation.

In addition to stem traits, leaf morphological and nutrient traits can also differ among these three growth forms (Zhu and Cao 2010, Asner and Martin 2012, Campanello et al. 2016, Werden et al. 2017). Lianas typically have higher specific leaf area (SLA) and foliar nutrient concentrations and lower leaf construction costs than co-occurring trees (Zhu and Cao 2010, Asner and Martin 2012, Wyka et al. 2013, Campanello et al. 2016, Werden et al. 2017). A few studies contradict this, as they showed no differences in SLA and leaf nitrogen and phosphorus concentrations between lianas and trees (van der Sande et al. 2013, Collins et al. 2016). A study also found that lianas have lower SLA than trees and found no difference in foliar nitrogen concentration between lianas and trees (Smith-Martin et al. 2019). Campanello et al. (2016) pointed out that lianas have higher SLA and lower leaf construction cost than scandent shrubs. Nevertheless, little information has been available to compare leaf morphological and nutrient traits among lianas, scandent shrubs and trees.

Here, we measured 16 traits associated with stem anatomy and hydraulics and leaf morphology and nutrients for eight lianas, eight scandent shrubs and 21 trees with evergreen and deciduous habits in a subalpine cold temperate forest in Yunnan, Southwest China. Three specific questions were addressed: (i) How do stem anatomical traits and leaf morphological and nutrient traits differ among the three coexisting growth forms? We predicted that lianas have higher values for vessel diameter, potential hydraulic conductivity, SLA, foliar nitrogen, phosphorus and potassium concentrations, and lower wood density, vessel density and leaf carbon concentration than trees, and scandent shrubs have intermediate values. (ii) How are key stem and leaf traits linked, and is there coordination or trade-offs among these three coexisting growth forms? We predicted that stem hydraulic conductivity would be positively associated with SLA, foliar nitrogen, phosphorus and potassium concentrations, and negatively with leaf carbon concentration, wood density and vessel density, reflecting trade-offs and coordination between stem and leaf traits. (iii) Is the divergence of stem or leaf traits mainly explained by growth form? We know that lianas, scandent shrubs and trees are apparently different in stem growth patterns, we therefore predicted that the divergence of growth forms is principally associated with stem trait variation.

## Materials and methods

### Study site and species

This study was carried out at the Lijiang Alpine Botanical Garden (27°00′N, 100°10′E; 2600–3200 m above sea level), Yunnan, Southwest China. The mean annual temperature at this location is 8.41 °C, and the mean annual precipitation is ca. 1100 mm, with distinct rainy (June–September) and dry seasons (October–May) (Figure S1 available as Supplementary data at *Tree Physiology* Online). The vegetation is subalpine coniferous and broadleaved mixed forest. The dominant species include coniferous species (e.g. *Pinus yunnanensis*), sclerophyllous broadleaved species (e.g. *Quercus* spp.) and rhododendron trees and shrubs. The soil type is a brown loam, with pH being 4.85–6.74. The total carbon and nitrogen contents of soil are 83.46 and 6.67 g kg^−1^, and NH_4_^+^ and NO_3_^−^ contents are 23.36 and 9.6 mg kg^−1^, respectively. We collected samples from eight liana, eight scandent shrub and 21 tree species in October 2017, encompassing 16 evergreen species and 21 deciduous species (Table 1). For each species, we sampled three individuals. We selected sunlit, fully expanded and healthy leaves from adult plants for trait measurements. In total, we measured 16 stem anatomical and hydraulic traits and leaf morphological and nutrient traits across all the study species (Table 2).

**Table 1:** Names, species code, family, growth form and leaf habit of 37 species.

Species	Code	Family	Growth form	Leaf habit
*Apios carnea* (Wall.) Benth. ex Baker	Ac	Fabaceae	L	D
*Aristolochia faucimaculata* H. Zhang & C. K. Hsien	Af	Aristolochiaceae	L	D
*Celastrus glaucophyllus* Rehd. et Wils.	Cg	Celastraceae	L	D
*Clematis subumbellata* Kurz	Cs	Ranunculaceae	L	D
*Holboellia latifolia* Wall.	Hl	Berberidaceae	L	E
*Lonicera acuminata* Wall.	La	Caprifoliaceae	L	E
*Sabia yunnanensis* Franch.	Sy	Sabiaceae	L	D
*Schisandra sphaerandra* Stapf	Ss	Schisandraceae	L	D
*Berchemia sinica* Schneid.	Bs	Rhamnaceae	SS	D
*Dalbergia hancei* Benth.	Dh	Fabaceae	SS	D
*Elaeagnus delavayi* Lecomte	Ed	Elaeagnaceae	SS	E
*Rosa cymosa* Tratt.	Rc	Rosaceae	SS	E
*Rosa longicuspis* Bertol.	Rl	Rosaceae	SS	E
*Rubus niveus* Thunb.	Rn	Rosaceae	SS	E
*Smilax ferox* Wall. ex Kunth	Sf	Smilacaceae	SS	E
*Smilax microphylla* C. H. Wright	Sm	Smilacaceae	SS	E
*Acer pectinatum* Wall. ex Nichols.	Ap	Sapindaceae	T	D
*Cornus walteri* Wangerin	Cw	Cornaceae	T	D
*Euonymus tingens* Wall.	Et	Celastraceae	T	E
*Hydrangea macrocarpa* Hand.-Mazz.	Hm	Hydrangeaceae	T	D
*Litsea chunii* Cheng	Lc	Lauraceae	T	D
*Lonicera setifera* Franch.	Ls	Caprifoliaceae	T	D
*Malus ombrophila* Hand.-Mazz.	Mo	Rosaceae	T	D
*Philadelphus calvescens* (Rehder) S. M. Hwang	Pc	Hydrangeaceae	T	D
*Piptanthus tomentosus* Franch.	Pt	Fabaceae	T	D
*Populus rotundifolia* var. *bonatii* (H. Léveillé) C. Wang et S. L. Tung	Pr	Salicaceae	T	D
*Quercus guajavifolia* H. Léveillé	Qg	Fagaceae	T	E
*Quercus senescens* Hand.-Mazz.	Qs	Fagaceae	T	E
*Rhododendron decorum* Franch.	Rd	Ericaceae	T	E
*Rhododendron oreotrephes* W. W. Smith	Ro	Ericaceae	T	E
*Rhododendron racemosum* Franch.	Rra	Ericaceae	T	E
*Rhododendron rubiginosum* Franch.	Rr	Ericaceae	T	E
*Rhododendron yunnanense* Franch.	Ry	Ericaceae	T	E
*Salix cathayana* Diels	Sc	Salicaceae	T	D
*Salix wallichiana* Anderss.	Sw	Salicaceae	T	D
*Sorbus hupehensis* C. K. Schneid.	Sh	Rosaceae	T	D
*Viburnum betulifolium* Batal.	Vb	Adoxaceae	T	D

Nomenclature follows Flora of China (http://foc.iplant.cn/). L = liana; SS = scandent shrub; T = tree; D = deciduous; E = evergreen.

**Table 2:** Overview of the stem and leaf traits and their abbreviations and units.

Trait	Abbreviation	Unit
Stem traits		
Hydraulically weighted vessel diameter	*D*_h_	μm
Maximum vessel diameter	*D*_max_	μm
Theoretical hydraulic conductivity	*K*_t_	kg m^−1^ s^−1^ MPa^−1^
Vessel cross-sectional area	VCA	%
Vessel density	VD	no. mm^−2^
Wood density	WD	g cm^−3^
Leaf traits		
Carbon concentration	C	g kg^−1^
Carbon nitrogen ratio	C/N	
Potassium concentration	K	g kg^−1^
Leaf density	LD	kg m^−3^
Leaf thickness	LT	μm
Nitrogen concentration	N	g kg^−1^
Nitrogen/phosphorus ratio	N/P	
Phosphorus concentration	P	g kg^−1^
Specific leaf area	SLA	cm^2^ g^−1^
Stable carbon isotope composition	δ^13^C	‰

### Stem anatomical and hydraulic traits

We collected terminal stems with diameters of ~1 cm from adult individuals to measure the sapwood density (WD, g cm^−3^) and other anatomical traits. The samples came from adult scandent shrubs at their lianescent stage. The fresh volume of a small sapwood sample was measured by displacement of water and then the sapwood was dried at 80 °C for 48 h to calculate sapwood density by dry mass/fresh volume.

For stem anatomical characteristics, formalin-alcohol-acetic acid (FAA)-fixed stem sections were cut with a microtome at 10 to 25 μm thickness, stained with 1% safranin, mounted on slides, and viewed under a light microscope (Leica Microsystems Ltd, Leica DM2500, Wetzlar, Germany). Each slide was photographed with a digital camera (2560 × 1920 pixels), with three to five images at 50×, 100×, 200× and 400× magnification. We processed photographs by sharpening them with Photoshop CS5 (Adobe Systems, San Jose, CA, USA) to better distinguish vessels from other tissues. Ten to 15 images were processed for each species. We then measured vessel area, major and minor axes and numbers with ImageJ software (National Institutes of Health, Bethesda, MD, USA). Vessel diameter was calculated according to Fan et al. (2012):

(1) \begin{equation*} {D}_i={\left(\frac{32{(ab)}^3}{a^2+{b}^2}\right)}^{\frac{1}{4}} \end{equation*}

where *a* and *b* represent the radius of the major and minor axis, respectively. *D*_h_ (μm) was the hydraulically weighted vessel diameter and was calculated as (Poorter et al. 2010):

(2) \begin{equation*} {D}_{\mathrm{h}}={\left[\left(\frac{1}{n}\right)\sum_{i=1}^n{D_i}^4\right]}^{\frac{1}{4}} \end{equation*}

Diameter of the biggest 10 vessels in the field of view for one individual was calculated as the maximum vessel diameter (*D*_max_, μm). Vessel density (VD, no. mm^−2^) was defined as the number of vessels per unit tissue area in the stem cross sections. The vessel cross-sectional area (VCA, %) was calculated as the percentage of cross-sectional area occupied by the vessels in the field of view. The theoretical hydraulic conductivity (*K*_t_, kg m^−1^ s^−1^ MPa^−1^) was calculated following the Hagen–Poiseuille principle (Tyree and Ewers 1991, Poorter et al. 2010):

(3) \begin{equation*} {K}_{\mathrm{t}}=\left(\frac{\pi \rho}{128\eta A}\right)\sum_{i=1}^n{D_i}^4 \end{equation*}

where *π* is the circular constant at 3.14, *ρ* is the density of water at 25 °C (997.05 kg m^−3^ at 25 °C), *η* is the viscosity of water at 25 °C (0.89 × 10^−9^ MPa s at 25 °C) and *A* is the area of the images. Generally, *K*_t_ is greater than the actual conductivity due to the resistance of the vessel cell walls and perforation plates (Sperry et al. 2005), and the fact that cavitated vessels are also not excluded. Here, we assume that the species ranking will not be significantly changed by these additional conditions and that the actual stem-specific xylem hydraulic conductivity scales positively with *K*_t_ (Poorter et al. 2010).

### Leaf morphological traits

We measured SLA(cm^2^ g^−1^) for each individual as the area of fresh leaves divided by their oven-dry mass. To determine this, each leaf was excised, and the petiole was removed. Individual leaflets were used for species with compound leaves. We used a scanner to scan fresh leaves at 300 d.p.i. resolution, and the ImageJ (National Institutes of Health) to measure the leaf area. Leaves were then oven-dried at 80 °C for at least 48 h to a constant value, and dry weight was measured with a balance with a precision of 0.0001 g (Mettler Toledo, AL204, Shanghai, China).

We measured leaf thickness (LT, μm) with paraffin-embedded leaf tissue sections. This process included the following steps: dehydration, wax immersion, embedding, sectioning to a thickness of 8–12 μm, safranin staining and sealing. We then used a light microscope (Leica Microsystems Ltd, Leica DM2500) with a mounted digital camera to take images at 50×, 100×, 200× and 400× magnification (2560 × 1920 pixels). Five images of each slide were taken at each magnification. We then used ImageJ software (National Institutes of Health) to determine the LT. Leaf density (LD, kg m^−3^) was calculated as 1/(SLA × LT).

### Leaf nutrient and carbon isotope analysis

Fresh leaf samples were oven-dried at 80 °C for at least 48 hours, ground to a fine powder with a crusher, and then passed through a 60-mesh sieve. The carbon (C, g kg^−1^) and nitrogen (N, g kg^−1^) concentrations were measured with a Dumas-type combustion C-N elemental analyzer (Vario MAX CN, Elementar Analysensysteme GmbH, Hanau, Germany), and phosphorus (P, g kg^−1^) and potassium (K, g kg^−1^) concentrations were measured with an inductively coupled plasma atomic-emission spectrometer (iCAP 7400, Thermo Fisher Scientific, Bremen, Germany). The C/N ratio and N/P ratio were then calculated.

After nutrient analysis, the remaining samples were passed through a 100-mesh sieve, and stable carbon isotopic composition (δ^13^C, ‰) was measured with an isotope ratio mass spectrometer (IsoPrime100, Isoprime Ltd, Cheadle, Manchester, UK), using Pee Dee Belemnite (PDB) as a standard. From the analysis, δ^13^C was calculated as follows:

(4) \begin{equation*} {\delta}^{13}\mathrm{C}\ \left({\mbox{\fontencoding{U}\char104}} \right)=\left[\left({R}_{\mathrm{sample}}\right)/\left({R}_{\mathrm{sample}}\right)-1\right]\times 1000 \end{equation*}

where *R*_sample_ and *R*_standard_ are the ratios of ^13^C/^12^C of sample and the PDB standard, respectively. The δ^13^C of leaves indicates the long-term water-use efficiency integrated over the lifetime of a leaf (Farquhar et al. 1989). Less negative δ^13^C values generally indicate higher time-integrated water-use efficiency (Farquhar et al. 1989).

### Statistical analysis

The average value for each species was used in the statistical analyses. All data were log_10_-transformed prior to statistical analyses to ensure normality and homoscedasticity. Linear mixed-effect models were used to evaluate the effects of growth form and leaf habit on trait variance with *lmer* function in the *lme4* package. We used growth form and leaf habit as fixed effects and species as a random effect. The results showed no significant interaction (*P* > 0.05) between growth form and leaf habit for all traits (data not shown), suggesting that growth form and leaf habit independently influenced variance in all traits. One-way analysis of variance (ANOVA) was then used to identify the differences in each trait among lianas, scandent shrubs and trees, and Tukey’s HSD post hoc tests were used to examine differences between these growth forms. Two-way multivariate analysis of variance (MANOVA) was performed to evaluate the variance explained by growth forms and leaf habits with the *manova* function from the package *stats*.

We used Pearson’s correlation analysis to test for bivariate relationships between pairs of traits among three growth forms. In addition, to further determine whether lianas, scandent shrubs and trees exhibited similar scaling relationships, we compared the differences in slopes, intercepts and shifts along a common slope among the three growth forms using a standardized major axis estimation with SMATR software (Warton et al. 2006).

A principal component analysis (PCA) was performed to evaluate how these traits were associated with one another. It was also used to compare the distributions of species in multivariate space. The factor loadings and scores of the first two principal components (PC 1 and PC 2) in the PCA were calculated to show the multivariate spatial associations of traits and species.

We constructed a phylogenetic tree of 37 species using *phylo.maker* function in the *V.PhyloMaker* package (Figure S2 available as Supplementary data at *Tree Physiology* Online). Blomberg’s *K* statistic was used to assay for phylogenetic signals of traits, and the tendency of related species to resemble one another (Blomberg et al. 2003). We found that all Blomberg’s *K* values were <1 (see Table S1 available as Supplementary data at *Tree Physiology* Online), indicating weak phylogenetic signals. In addition, we did phylogenetic ANOVA to exclude the phylogenetic effect on differences in stem and leaf traits among growth forms with *aov.phylo* function in the *geiger* package.

One-way ANOVA, Pearson’s correlation and PCA analyses were carried out with SPSS (version 20.0; SPSS, Inc., Chicago, IL, USA). Other analyses were performed in R software (version 4.0.3; R Development Core Team 2020).

## Results

### Comparison of stem and leaf traits among lianas, scandent shrubs and trees

**Figure 1.**
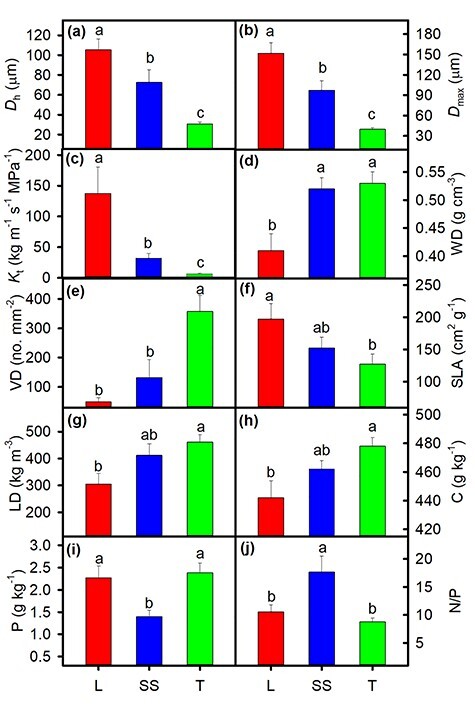
Differences in means (±SE) of hydraulically weighted vessel diameter (*D*_h_; a), maximum vessel diameter (*D*_max_; b), theoretical hydraulic conductivity (*K*_t_; c), sapwood density (WD; d), vessel density (VD; e), SLA (f), LD (g), carbon concentration (C; h), phosphorus concentration (P; i) and N/P ratio (j) among eight liana (L), eight scandent shrub (SS) and 21 tree (T) species. Different letters above bars indicate significant differences (*P* < 0.05) among groups according to one-way ANOVA (Tukey’s HSD).

For stem traits, the rank order of the significant differences in mean hydraulically weighted vessel diameter (*D*_h_) (105.3 ± 11.2 > 72.5 ± 12.8 > 30.6 ± 2 μm), maximum vessel diameter (*D*_max_) (151.8 ± 15.5 > 97.2 ± 14.1 > 40.0 ± 2.6 μm) and theoretical hydraulic conductivity (*K*_t_) (137.6 ± 42.8 > 31.9 ± 8 > 6.6 ± 0.8 kg m^−1^ s^−1^ MPa^−1^) was lianas > scandent shrubs > trees (all *P <* 0.001) (Figure 1). The rank order for sapwood density (WD) and vessel density (VD) among growth forms was lianas < scandent shrubs = trees (*P* = 0.003) and lianas = scandent shrubs < trees (*P* < 0.001), respectively. For leaf traits, lianas had a significantly higher SLA and lower LD and carbon concentration (C) than trees (*P =* 0.031, *P =* 0.016 and *P =* 0.015, respectively). The values for SLA, LD and C of scandent shrubs were intermediate between those of the trees and lianas but, were not significantly different from those of either growth form. Unexpectedly, scandent shrubs had a lower phosphorus concentration (P; *P* = 0.011) and a higher nitrogen/phosphorus ratio (N/P; *P* < 0.001) than lianas and trees, whereas lianas, and trees did not differ in P or N/P ratio. There were no significant differences among lianas, scandent shrubs and trees in terms of LT, N concentration, potassium concentration (K), C/N ratio, stable carbon isotopic composition (δ^13^C) or vessel cross-sectional area (VCA) (see Figure S3 available as Supplementary data at *Tree Physiology* Online). After phylogenetic correction, differences in LD, C and P became marginally significant, and only the difference in SLA became nonsignificant among three growth forms (see Table S2 available as Supplementary data at *Tree Physiology* Online).

### Functional trait bivariate relationships

**Figure 2.**
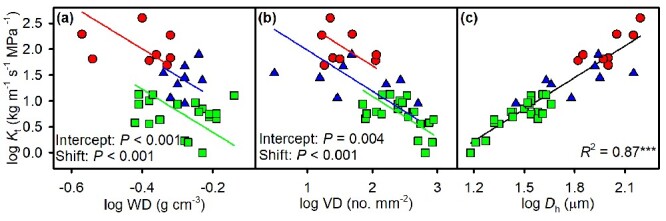
The log–log relationships of stem theoretical hydraulic conductivity (*K*_t_) with (a) wood density (WD), (b) VD and (c) hydraulic weighted vessel diameter (*D*_h_) across eight lianas (red circles), eight scandent shrubs (blue triangles) and 21 trees (green squares). See Table 3 for regression statistics.

**Figure 3.**
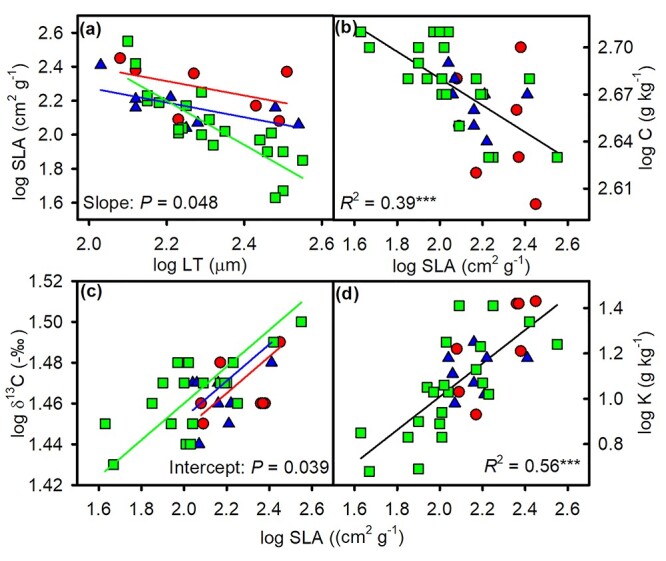
The log–log relationships of SLA with (a) LT, (b) foliar carbon concentration (C), (c) stable carbon isotopic composition (δ^13^C) and (d) potassium concentration (K) across eight lianas (red circles), eight scandent shrubs (blue triangles) and 21 trees (green squares). See Table 3 for regression statistics.

For stem traits, there were significantly negative relationships between *K*_t_ and VD, and the behavior of *K*_t_ in relation to WD was similar but much weaker (*R*^2^ = 0.52, *P* < 0.001; *R*^2^ = 0.2, *P* = 0.005; respectively; Figure 2 and Table S3 available as Supplementary data at *Tree Physiology* Online), with a common slope but different intercepts and shifts among the three growth forms (Table 3). *K*_t_ and *D*_h_ showed highly significant positive relationships (*R*^2^ = 0.87, *P* < 0.001), with a single slope and intercept across the three growth forms, but lianas showed a shift towards higher values for both traits (*P* < 0.001). For leaf traits, across all species, SLA was significantly negatively related to LT, with significantly different slopes between scandent shrubs and trees (*P* = 0.048; Figure 3 and Table 3). The significant C–SLA and δ^13^C–SLA relationships were described by a common slope, with significantly different shifts along the common slope in the C–SLA relationship (*P* = 0.019) and significantly different intercepts in the δ^13^C–SLA relationship among three growth forms (*P <* 0.05). There was a highly significant, positive relationship between K and SLA (*R*^2^ = 0.56, *P <* 0.001), with a single slope and intercept among the three growth forms but a different shift along the common slope (*P* = 0.035).

**Table 3:** Tests of the slope, intercept and shift along a common slope for bivariate relationships among the three growth forms (L for liana, SS for scandent shrub and T for trees; Table 1) using standardized major axis regression analyses as implemented within SMATR software.

*y* ~ *x*	Slope				Intercept				Shifts along the common slope (*P*)
	L	SS	T	*P*	L	SS	T	*P*	
log *K*_t_ ~ log WD	−4.17			0.191	0.34^a^	0.23^a^	−0.44^b^	**<0.001**	**<0.001**
log *K*_t_ ~ log VD	−0.79			0.222	3.26^a^	2.77^ab^	2.67^b^	**0.004**	**<0.001**
log *K*_t_ ~ log *D*_h_	2.09			0.337	−2.17	−2.37	−2.32	0.358	**<0.001**
log SLA ~ log LT	−0.88^ab^	−0.67^a^	−1.55^b^	**0.048**					
log C ~ log SLA	−0.13			0.472	2.95	2.95	2.95	0.937	**0.019**
log δ^13^C ~ log SLA	0.09			0.84	1.27^b^	1.27^ab^	1.28^a^	**0.039**	0.235
log K ~ log SLA	1.02			0.573	−1.07	−1.08	−1.04	0.824	**0.035**
log SLA ~ log WD	−2.26			0.253	1.36	1.52	1.41	0.145	**0.015**
log C ~ log *K*_t_	−0.08			0.339	2.80^a^	2.78^a^	2.74^b^	**0.002**	**<0.001**
log SLA ~ log *K*_t_	0.62			0.427	1.06^b^	1.29^b^	1.59^a^	**0.003**	**<0.001**
log K ~ log *K*_t_	0.63			0.191	−0.07^b^	0.24^b^	0.58^a^	**<0.001**	**<0.001**

Significant differences (*P* ≤ 0.05) are indicated in bold. Different lowercase letters identify growth forms that significantly differed in post hoc tests. See Table 2 for trait abbreviations. The unit of δ^13^C was converted to positive when calculating log δ^13^C ~ log SLA relationship.

**Figure 4.**
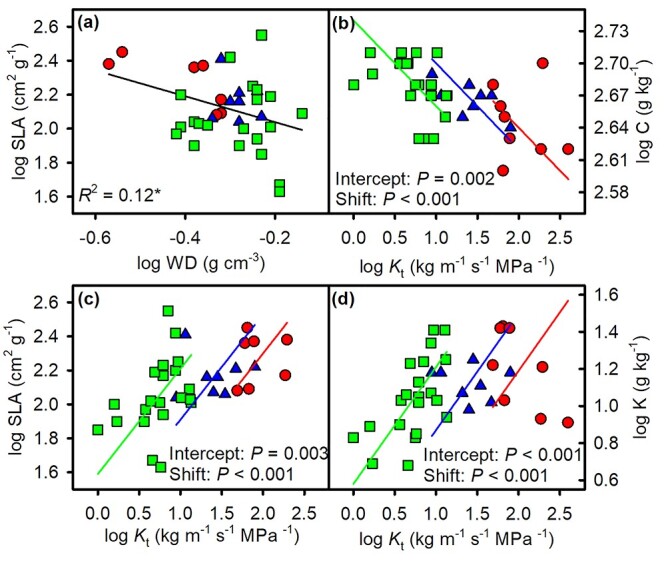
The log–log relationships (a) between SLA and wood density (WD) and the relationships of theoretical hydraulic conductivity (*K*_t_) with (b) foliar carbon concentration (C), (c) SLA and (d) potassium concentration (K) across eight lianas (red circles), eight scandent shrubs (blue triangles) and 21 trees (green squares). See Table 3 for regression statistics.

Considering both the stem and leaf traits, the SLA–WD and C–*K*_t_ relationships were significantly negative (Figure 4 and Table S3 available as Supplementary data at *Tree Physiology* Online), and both had the same slope for each of the three growth forms, with significantly different intercepts and shifts among three growth forms (Table 3). Specific leaf area and K had a significantly positive correlation with *K*_t_ (Figure 4). The two relationships did not significantly differ in terms of slope, although the intercepts and shifts significantly differed among the three growth forms (Table 3).

### Trait affinities of lianas, scandent shrubs and trees

**Figure 5.**
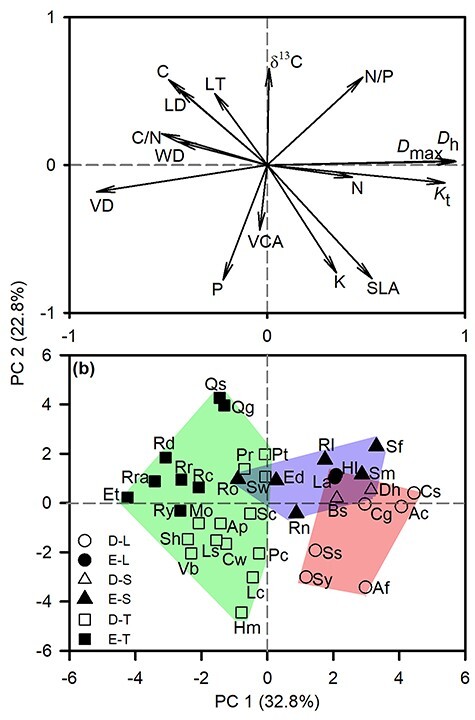
Position of (a) traits and (b) species on first two axes of principal component analysis for all traits included in this study. In (b) lines were drawn around groups of species with different growth forms. D-L: deciduous lianas; E-L: evergreen lianas; D-S: deciduous scandent shrubs; E-S: evergreen scandent shrubs, D-T: deciduous trees; E-T: evergreen trees. Species codes were shown in Table 1. See Table 2 for trait abbreviations.

**Figure 6.**
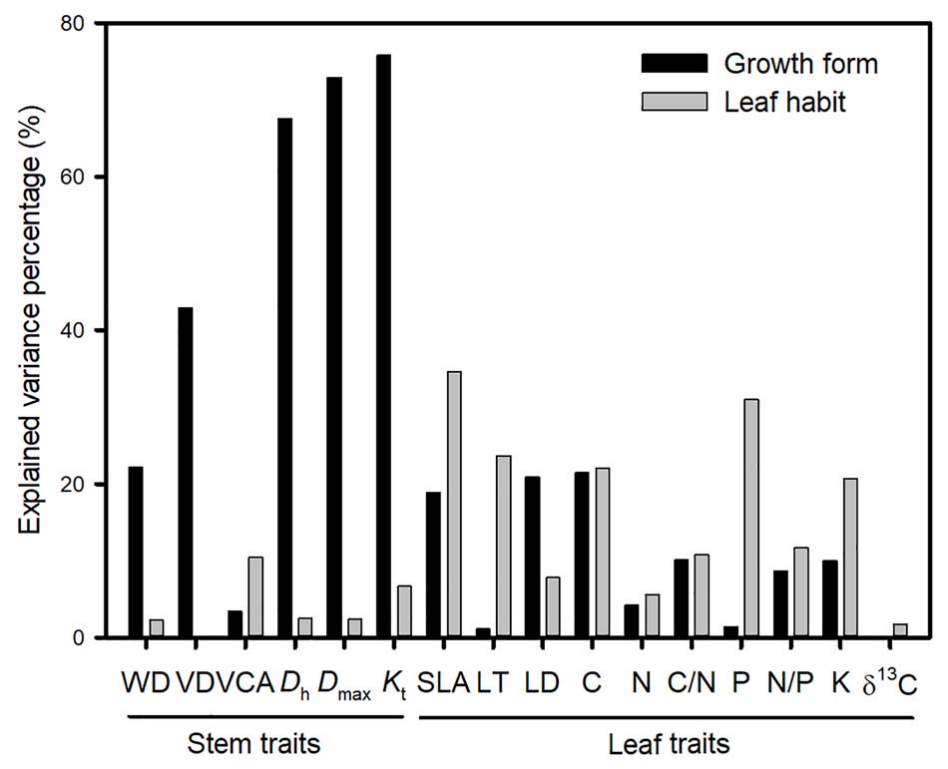
The fraction of variance in stem and leaf traits explained by growth form and leaf habit based on two-way MANOVA. See Table 2 for trait abbreviations.

The first two axes of the PCA explained 32.8 and 22.8% of the total variance in the 16 traits, respectively (Figure 5). The first axis (PC1) was primarily related to stem traits and showed a trade-off between traits related to hydraulic efficiency (*D*_h_ and *K*_t_) and those related to hydraulic safety (VD and WD), with a greater percentage of variance explained by growth form than leaf habit (Figure 6 and Table S4 available as Supplementary data at *Tree Physiology* Online). The second axis (PC2) was mainly related to leaf traits and showed a trade-off between traits related to conservative strategies (δ^13^C, LT and C) and those related to fast resource acquisition (P, SLA and K) (Figure 5), with a higher percentage of variance explained by leaf habit and less by growth form (Figure 6 and Table S4 available as Supplementary data at *Tree Physiology* Online). In terms of trait clusters, the three growth forms were separately grouped along the stem trait axis (PC 1). The lianas were grouped together in the lower right corner and associated with high SLA, K, *D*_h_ and *K*_t_. The trees were grouped on the left and associated with high LT, C, VD and WD. Scandent shrubs were grouped between lianas and trees (Figure 5).

## Discussion

This study, to our knowledge, is the first attempt to investigate the differentiation of functional strategies in terms of stem and leaf traits among coexisting lianas, scandent shrubs and trees. We found that lianas, scandent shrubs and trees follow a fast–slow continuum mediated by stem and leaf traits, and scandent shrubs showed intermediate values and partially overlapped with lianas and trees. It seems that these differences among lianas, scandent shrubs and trees drive stem trait variation. Additionally, the deciduous liana species were associated with the fast end and evergreen tree species with the slow end of the growth strategy continuum.

### Lianas, scandent shrubs and trees follow a fast–slow growth trait contrast mediated by stem and leaf traits

Among the combined traits that were measured, lianas trended towards being acquisitive in the sense of being fast-growing and resource requiring, while trees behaved more conservatively with regard to resources and growth (Markesteijn et al. 2011, Collins et al. 2016). Scandent shrubs showed intermediate values with overlaps between lianas and trees. In terms of traits related to stem hydraulic efficiency, we found an obvious pattern of lianas > scandent shrubs > trees (Figure 1). Wide conduits allow species to achieve higher hydraulic efficiency because higher hydraulic efficiency is calculated by conduit diameter (Poorter et al. 2010, Zanne et al. 2010, van der Sande et al. 2019). The greater vessel diameter and potential hydraulic conductivity in lianas mean higher water transport efficiency that contributes to fast resource acquisition, growth and tissue turnover (Zhu and Cao 2009). In contrast, coexisting trees had higher sapwood density and vessel density, suggesting that trees are more conservative and stress-resistant and have a slow growth and tissue turnover (Markesteijn et al. 2011, van der Sande et al. 2019). Furthermore, we found that scandent shrubs were similar to trees and higher than lianas in terms of sapwood density. Lianas invest more resources into fast stem elongation and efficient water transport systems, while scandent shrubs allocate more biomass into self-support tissue in their seedling stage (Campanello et al. 2016). A scandent shrub had stiffer stems than a liana based on the comparison of stem mechanical traits at both juvenile and mature stages (Chen et al. 2014), and scandent shrubs were similar in biomechanical properties (such as stem flexibility) and in anatomical characteristics (such as xylem tissue proportions) to tree saplings (Kennard 1998). Scandent shrubs might strengthen their resistance to freezing conditions through increased sapwood density (Lahaye et al. 2005, Wagner et al. 2012).

Specific leaf area was significantly higher in lianas than in trees, with scandent shrubs being intermediate between lianas and trees. High SLA in lianas was related to light capture or fast resource acquisition (Collins et al. 2016), because leaves with high SLA have shorter diffusion paths from stomata to chloroplasts, resulting in a higher mesophyll conductance and photosynthetic assimilation (Wright et al. 2004, Onoda et al. 2017). We did not find significant differences in leaf N, P and K concentrations between lianas and trees, inconsistent with other studies in tropical forests (Zhu and Cao 2010, Asner and Martin 2012). Furthermore, the N/P ratio of lianas and trees was lower than 14 (Figure 1), which indicates that N might be a limiting nutrient for lianas and trees at this high elevation (see Koerselman and Meuleman 1996, Du et al. 2020). Consistent with this, Reich and Oleksyn (2004) concluded that N/P ratio decreases with mean temperature and N is the major limiting nutrient in temperate and high-latitude soils. However, the N/P ratio of scandent shrubs reached up to 17, which suggests to some extent that scandent shrubs had a P deficit. In fact, the P concentration of scandent shrubs indeed was low (Figure 1). We call for more comparative studies on ecological adaptation strategies of lianas and scandent shrubs in forest ecosystems at high elevation.

### Coordination of stem and leaf traits across lianas, scandent shrubs and trees

Leaves capture light and acquire carbon, while stems transport water and nutrients and mechanically support the leaves, thus we expected that stem and leaf traits are highly coordinated (Westoby et al. 2002, Zhang and Cao 2009). We indeed found that SLA and leaf K concentration were positively correlated with theoretical hydraulic conductivity across these three growth forms (Figure 4). This suggests that effective stem xylem transport could mediate leaf acquisitive traits, e.g. SLA (Santiago et al. 2004, Zhang and Cao 2009, Zhu and Cao 2009, Nolf et al. 2015).

We found a significant correlation between K and many other traits (Table S3 available as Supplementary data at *Tree Physiology* Online). Although there are only a few studies on associations between nutrient concentrations and stem hydraulic traits (but see Zhu and Cao 2009, Werden et al. 2017), previous studies have suggested that K is an important dissolved ion in cell sap, contributing to cell osmoregulation and stomatal control (Wright et al. 2005, Salzer et al. 2006, Santiago and Wright 2007). Additionally, K replacement by hexose for osmotic control reduces the demands for carbon as a substrate (Sharp et al. 1990). Our above results might indicate that K is an important nutrient in the subalpine cold temperate forest. Wright et al. (2005) have pointed out that K should be considered to be an important part of the leaf economics spectrum, if K is tightly correlated with SLA, N and P. Based on our findings, close associations of leaf K with other traits related to leaf economics spectrum should be further investigated (Wright et al. 2005).

For the three different growth forms, the relationship between pairs of traits was complicated. We found that more significant correlations occur in trees than lianas and scandent shrubs (Table S3 available as Supplementary data at *Tree Physiology* Online). Although the three growth forms diverged in their positions along the regression lines, the slopes of trait relationships across lianas, scandent shrubs and trees were similar (Figures 2−4 and Table 3), suggesting that these trait relationships are convergent across the growth forms. This pattern is consistent with previous studies on functional trait relationships across functional groups (Wright et al. 2005). This might reflect that different species are arranged in the adaptive or competitive positions along an ecological strategy axis (Wright et al. 2005).

### Effects of growth form and leaf habit on trait variation among lianas, scandent shrubs and trees

We found that coexisting lianas, scandent shrubs and trees followed a fast–slow continuum along the first axis of PCA (Figure 5), which could be defined as the stem economics spectrum that reflected a trade-off between hydraulic efficiency and wood resistance (Chave et al. 2009). Stem traits could drive the divergence of liana, scandent shrub and tree growth forms (Figure 6 and Table S4 available as Supplementary data at *Tree Physiology* Online). Dias et al. (2019) also pointed out that the change in vascular strategy (variation in vessel lumen fraction and vessel composition) makes lianas hydraulically distinctive from trees. In addition to growth form, trait variation can also be related to leaf habit, e.g. deciduous vs evergreen (Eamus 1999, Fu et al. 2012, Zhang et al. 2013). We found that deciduous and evergreen species were grouped along the second axis of PCA (Figure 5), which represented the leaf economic spectrum and reflected a trade-off: resource acquisition vs construction cost (Wright et al. 2004, Zhu and Cao 2010, Osnas et al. 2013). Leaf habit explained a greater variance in leaf traits than growth forms (Figure 6 and Table S4 available as Supplementary data at *Tree Physiology* Online). Previous studies proved that deciduous species have higher leaf nitrogen and phosphorus concentrations, SLA and higher photosynthesis than evergreen species, employing a more acquisitive strategy (Eamus 1999, Fu et al. 2012, Bai et al. 2015). Together, although leaf habit explained a greater variance in leaf traits, in our case total trait variation was principally associated with growth form rather than leaf habit.

Interestingly, we found that deciduous lianas tended to be distributed at the fast end of the trait spectrum, and evergreen trees at the slow end of the spectrum (Figure 5). High SLA and low leaf carbon cost mediated by deciduous habit were coupled with the efficient stem hydraulic system mediated by the large vessel diameters, which might allow subalpine temperate lianas to have potentially maximum competitive and growth advantages during frost-free periods. This suggests that deciduous leaf habit for these lianas from subalpine forests may be an important adaptive strategy in frost-prone habitats (Jiménez-Castillo and Lusk 2013, Fang et al. 2017).

We also found that two evergreen scandent shrubs overlapped with three deciduous trees and two deciduous scandent shrubs overlapped with two evergreen lianas (Figure 5). This demonstrates that variation in the traits associated with growth forms is attributed to leaf habit, though stem hydraulic properties in particular were strongly associated with growth form (Figure 6). Chen et al. (2017) have reported that lianas had similar hydraulic properties to deciduous trees. This suggests that the combination of growth form and leaf habit could produce an overlapping functional strategy, though we did not find an interaction between growth form and leaf habit. Trait associations between growth forms and leaf habits for lianas, including scandent shrubs in subalpine forests, as well as other forest types should be further investigated to better understand how climbing plants succeed in different forest ecosystems.

## Conclusions

This study investigated the differentiation in functional strategy in terms of stem and leaf traits among coexisting lianas, scandent shrubs and trees in a subalpine cold temperate forest in Southwest China. We found that lianas, scandent shrubs and trees follow a fast–slow continuum along stem and leaf traits, with the deciduous liana species associated with the fast end and evergreen tree species with the slow end of the growth strategy continuum. We also found that the variation in stem traits among lianas, scandent shrubs and trees was mainly associated with growth forms, while variation in leaf traits was mainly related to leaf habit. Because work on functional traits and differences among lianas and scandent shrubs is still lacking, we call for more studies on key functional traits of lianas, as well as scandent shrubs, in regions outside the tropics.

## Supplementary Material

Supplementary_Data_R1_tpab049
