# Supplementary material for: Differentiation in stem and leaf traits among sympatric lianas, scandent shrubs and trees in a subalpine cold temperate forest
Source: Tree Physiol. 2021 Apr 5;41(11):1992–2003. doi: 10.1093/treephys/tpab049 (PMC8597974; doi:10.1093/treephys/tpab049)
Supplement: Supplementary_Data_R1_tpab049 [file supplementary_data_r1_tpab049.docx]

**Supplementary Data**

**Differentiation in stem and leaf traits among sympatric lianas, scandent shrubs and trees in a subalpine cold temperate forest**

Ke-Yan Zhang^1,2,3*^, Da Yang^1,2*^, Yun-Bing Zhang^1,2,3*^, David S. Ellsworth^4^, Kun Xu^5^, Yi-Ping Zhang^1,2^, Ya-Jun Chen^1,2^, Fangliang He^6^, Jiao-Lin Zhang^1,2,7^

^1^CAS Key Laboratory of Tropical Forest Ecology, Xishuangbanna Tropical Botanical Garden, Chinese Academy of Sciences, Mengla, Yunnan 666303, China; ^2^Center of Plant Ecology, Core Botanical Gardens, Chinese Academy of Sciences, Mengla, Yunnan 666303, China; ^3^University of Chinese Academy of Sciences, Beijing 100049, China; ^4^Hawkesbury Institute for the Environment, Western Sydney University, Locked Bag 1797, Penrith NSW 2751 Australia; ^5^Lijiang Forest Ecosystem Research Station, Kunming Institute of Botany, Chinese Academy of Sciences, Lijiang 674100, China; ^6^Department of Renewable Resources, University of Alberta, Edmonton, Alberta T6G 2H1, Canada; ^7^Corresponding author (zjl@xtbg.org.cn)

Full address of the corresponding author:

Prof. Dr. Jiao-Lin Zhang

CAS Key Laboratory of Tropical Forest Ecology, Xishuangbanna Tropical Botanical

Garden, Chinese Academy of Sciences, Mengla, Yunnan 666303, China

Email: zjl@xtbg.org.cn

Tel.: +86-691-8713046

Fax: +86-691-8715070

*These authors contributed equally to this work.

**Table S1**. Blomberg’s *K* and *P* values for stem and leaf traits.

| Trait | Abbreviation | *K* | *P* |
| --- | --- | --- | --- |
| Wood density | WD | 0.038 | 0.123 |
| Vessel density | VD | 0.052 | 0.090 |
| Vessel cross-sectional area | VCA | 0.016 | 0.277 |
| Hydraulically weighted vessel diameter | *D*_h_ | 0.268 | **0.001** |
| Maximum vessel diameter | *D*_max_ | 0.447 | **0.002** |
| Theoretical hydraulic conductivity | *K*_t_ | 0.194 | **0.004** |
| Specific leaf area | SLA | 0.020 | 0.255 |
| Leaf thickness | LT | 0.011 | 0.308 |
| Leaf density | LD | 0.299 | **0.001** |
| [Carbon concentration](file:///D:\\Program%20Files\\Youdao\\Dict\\7.5.1.0\\resultui\\dict\\%3fkeyword=carbon) | C | 0.477 | **0.002** |
| Nitrogen concentration | N | 0.020 | 0.222 |
| [C/N ratio](file:///D:\\Program%20Files\\Youdao\\Dict\\7.5.1.0\\resultui\\dict\\%3fkeyword=carbon) | C/N | 0.022 | 0.204 |
| Phosphorus concentration | P | 0.018 | 0.225 |
| N/P ratio | N/P | 0.047 | 0.089 |
| Potassium concentration | K | 0.011 | 0.351 |
| Stable carbon isotope composition | δ^13^C | 0.802 | **0.001** |

Significant phylogenetic signals were in bold.

**Table S2.** The sum of squares, *F* values, and *P* values calculated by traditional one-way and phylogenetic ANOVAs for stem and leaf traits among three growth forms.

| Traits | Sum of squares | *F* | *P* | *Phylogenetic P* |
| --- | --- | --- | --- | --- |
| WD | 10.38 | 6.88 | **0.003** | **0.042** |
| VD | 16.85 | 14.96 | **<0.001** | **0.003** |
| VCA | 3.37 | 1.76 | 0.188 | 0.390 |
| *D*_h_ | 24.78 | 37.57 | **<0.001** | **0.001** |
| *D*_max_ | 26.68 | 48.69 | **<0.001** | **0.001** |
| *K*_t_ | 27.29 | 53.27 | **<0.001** | **0.001** |
| SLA | 7.24 | 4.28 | **0.022** | 0.112 |
| LT | 0.96 | 0.47 | 0.632 | 0.779 |
| LD | 8.61 | 5.34 | **0.010** | 0.053 |
| C | 7.90 | 4.78 | **0.015** | 0.077 |
| N | 3.68 | 1.94 | 0.160 | 0.361 |
| C/N | 5.49 | 3.06 | 0.060 | 0.205 |
| P | 8.36 | 5.14 | **0.011** | 0.068 |
| N/P | 12.64 | 9.20 | **0.001** | **0.011** |
| K | 3.60 | 1.89 | 0.167 | 0.352 |
| δ^13^C | 0.12 | 0.06 | 0.946 | 0.971 |

Significant difference (*P* < 0.05) was in bold. See Table S1 for trait abbreviations.

**Table S3.** Coefficients of Pearson’s correlation between pairs of stem and leaf traits.

**All species:**

|  | WD | VD | VCA | *D*_h_ | *D*_max_ | *K*_t_ | SLA | LT | LD | C | N | C/N | P | N/P | K |
| --- | --- | --- | --- | --- | --- | --- | --- | --- | --- | --- | --- | --- | --- | --- | --- |
| VD | 0.222 |  |  |  |  |  |  |  |  |  |  |  |  |  |  |
| VCA | −0.290 | **0.451^**^** |  |  |  |  |  |  |  |  |  |  |  |  |  |
| *D*_h_ | **−0.368^*^** | **−0.925^***^** | −0.081 |  |  |  |  |  |  |  |  |  |  |  |  |
| *D*_max_ | **−0.374^*^** | **−0.895^***^** | −0.031 | **0.993^***^** |  |  |  |  |  |  |  |  |  |  |  |
| *K*_t_ | **−0.450^**^** | **−0.723^***^** | 0.283 | **0.931^***^** | **0.947^***^** |  |  |  |  |  |  |  |  |  |  |
| SLA | **−0.345^*^** | −0.273 | 0.256 | **0.418^*^** | **0.434^**^** | **0.508^**^** |  |  |  |  |  |  |  |  |  |
| LT | 0.255 | 0.031 | −0.307 | −0.156 | −0.177 | −0.258 | **−0.655^***^** |  |  |  |  |  |  |  |  |
| LD | 0.186 | **0.367^*^** | −0.001 | **−0.417^*^** | **−0.409^*^** | **−0.409^*^** | **−0.650^***^** | −0.138 |  |  |  |  |  |  |  |
| C | 0.067 | **0.413^*^** | −0.161 | **−0.521^***^** | **−0.530^***^** | **−0.557^***^** | **−0.624^***^** | 0.323 | **0.537^***^** |  |  |  |  |  |  |
| N | −0.214 | −0.152 | 0.017 | 0.178 | 0.2 | 0.186 | 0.29 | **−0.329^*^** | −0.062 | −0.035 |  |  |  |  |  |
| C/N | 0.219 | 0.236 | −0.075 | −0.296 | −0.321 | −0.319 | **−0.420^*^** | **0.389^*^** | 0.184 | 0.273 | **−0.969^***^** |  |  |  |  |
| P | 0.022 | 0.216 | 0.191 | −0.163 | −0.173 | −0.092 | **0.385^*^** | −0.183 | −0.288 | **−0.360^*^** | 0.056 | −0.129 |  |  |  |
| N/P | −0.190 | −0.305 | −0.141 | 0.287 | 0.311 | 0.234 | −0.126 | −0.016 | 0.152 | 0.253 | **0.531^***^** | **−0.458^**^** | **−0.809^***^** |  |  |
| K | −0.218 | −0.160 | 0.292 | 0.292 | 0.292 | **0.373^*^** | **0.745^***^** | **−0.378^*^** | **−0.566^***^** | **−0.451^**^** | 0.27 | **−0.366^*^** | **0.452^**^** | −0.192 |  |
| δ^13^C | 0.13 | −0.069 | −0.038 | 0.081 | 0.096 | 0.079 | **−0.598^***^** | 0.289 | **0.505^**^** | 0.324 | −0.036 | 0.106 | −0.272 | 0.2 | **−0.410^*^** |

**Liana species:**

| Liana | WD | VD | VCA | *D*h | *D*max | *K*t | SLA | LT | LD | C | N | C/N | P | N/P | K |
| --- | --- | --- | --- | --- | --- | --- | --- | --- | --- | --- | --- | --- | --- | --- | --- |
| VD | −0.196 |  |  |  |  |  |  |  |  |  |  |  |  |  |  |
| VCA | −0.373 | 0.624 |  |  |  |  |  |  |  |  |  |  |  |  |  |
| *D*h | −0.020 | **−0.808^*^** | −0.059 |  |  |  |  |  |  |  |  |  |  |  |  |
| *D*max | −0.031 | **−0.733^*^** | 0.046 | **0.988^***^** |  |  |  |  |  |  |  |  |  |  |  |
| *K*t | −0.200 | −0.273 | 0.565 | **0.785^*^** | **0.846^**^** |  |  |  |  |  |  |  |  |  |  |
| SLA | −0.752 | 0.678 | **0.768^*^** | −0.440 | −0.399 | 0.135 |  |  |  |  |  |  |  |  |  |
| LT | **0.742^*^** | 0.051 | −0.279 | −0.229 | −0.265 | −0.313 | −0.482 |  |  |  |  |  |  |  |  |
| LD | −0.204 | −0.521 | −0.441 | 0.426 | 0.442 | 0.036 | −0.331 | −0.663 |  |  |  |  |  |  |  |
| C | −0.107 | 0.152 | −0.058 | −0.159 | −0.120 | −0.138 | −0.227 | 0.073 | 0.281 |  |  |  |  |  |  |
| N | **−0.875^**^** | 0.143 | 0.441 | 0.164 | 0.213 | 0.378 | 0.525 | **−0.707^*^** | 0.356 | 0.462 |  |  |  |  |  |
| C/N | **0.926^***^** | −0.102 | −0.526 | −0.263 | −0.309 | −0.504 | −0.659 | **0.801^*^** | −0.304 | −0.174 | **−0.952^***^** |  |  |  |  |
| P | −0.245 | 0.686 | 0.183 | **−0.745^*^** | **−0.757^*^** | −0.511 | **0.791^*^** | 0.192 | −0.727 | −0.248 | −0.109 | 0.049 |  |  |  |
| N/P | −0.364 | −0.404 | 0.094 | 0.603 | 0.631 | 0.545 | −0.192 | −0.541 | 0.717 | 0.514 | 0.692 | −0.600 | −0.781^*^ |  |  |
| K | −0.361 | **0.738^*^** | 0.122 | **−0.881^**^** | **−0.901^**^** | −0.678 | 0.745 | 0.031 | −0.446 | 0.078 | 0.110 | −0.061 | **0.851^**^** | −0.502 |  |
| δ13C | 0.264 | 0.089 | 0.258 | 0.150 | 0.233 | 0.338 | −0.399 | 0.078 | 0.163 | 0.608 | 0.153 | 0.005 | −0.571 | 0.529 | −0.420 |

**Scandent shrubs:**

|  | WD | VD | VCA | *D*h | *D*max | *K*t | SLA | LT | LD | C | N | C/N | P | N/P | K |
| --- | --- | --- | --- | --- | --- | --- | --- | --- | --- | --- | --- | --- | --- | --- | --- |
| VD | 0.583 |  |  |  |  |  |  |  |  |  |  |  |  |  |  |
| VCA | **0.806^*^** | **0.895^**^** |  |  |  |  |  |  |  |  |  |  |  |  |  |
| *D*h | −0.345 | **−0.947^***^** | **−0.708^*^** |  |  |  |  |  |  |  |  |  |  |  |  |
| *D*max | −0.309 | **−0.903^**^** | −0.658 | **0.977^***^** |  |  |  |  |  |  |  |  |  |  |  |
| *K*t | 0.339 | −0.422 | 0.018 | 0.690 | **0.727^*^** |  |  |  |  |  |  |  |  |  |  |
| SLA | −0.190 | −0.223 | −0.302 | 0.166 | 0.297 | −0.008 |  |  |  |  |  |  |  |  |  |
| LT | −0.130 | −0.492 | −0.387 | 0.474 | 0.294 | 0.213 | −0.641 |  |  |  |  |  |  |  |  |
| LD | 0.470 | **0.871^**^** | **0.831^*^** | **−0.768^*^** | −0.644 | −0.205 | −0.090 | −0.691 |  |  |  |  |  |  |  |
| C | −0.100 | 0.288 | 0.013 | −0.452 | −0.504 | −0.653 | −0.366 | 0.096 | 0.175 |  |  |  |  |  |  |
| N | −0.429 | 0.228 | −0.118 | −0.466 | −0.506 | **−0.788^*^** | 0.292 | −0.269 | −0.001 | 0.118 |  |  |  |  |  |
| C/N | 0.404 | −0.222 | 0.098 | 0.441 | 0.478 | **0.731^*^** | −0.320 | 0.284 | 0.004 | −0.022 | **−0.995^***^** |  |  |  |  |
| P | −0.038 | 0.189 | 0.131 | −0.192 | −0.169 | −0.098 | −0.092 | −0.021 | 0.178 | −0.407 | 0.211 | −0.253 |  |  |  |
| N/P | −0.376 | 0.106 | −0.179 | −0.323 | −0.373 | −0.670 | 0.334 | −0.253 | −0.098 | 0.336 | **0.812^*^** | −0.785^*^ | −0.398 |  |  |
| K | −0.224 | −0.264 | −0.331 | 0.144 | 0.027 | −0.177 | 0.257 | 0.231 | −0.584 | −0.219 | 0.613 | −0.637 | −0.149 | 0.665 |  |
| δ13C | 0.560 | 0.108 | 0.328 | 0.090 | 0.149 | 0.489 | −0.320 | 0.118 | 0.252 | 0.034 | **−0.861^**^** | **0.864^**^** | 0.069 | −0.853**^**^** | −0.781**^*^** |

**Tree species:**

| Tree | WD | VD | VCA | *D*h | *D*max | *K*t | SLA | LT | LD | C | N | C/N | P | N/P | K |
| --- | --- | --- | --- | --- | --- | --- | --- | --- | --- | --- | --- | --- | --- | --- | --- |
| VD | −0.227 |  |  |  |  |  |  |  |  |  |  |  |  |  |  |
| VCA | −0.396 | **0.629^**^** |  |  |  |  |  |  |  |  |  |  |  |  |  |
| *D*h | 0.055 | **−0.885^***^** | −0.197 |  |  |  |  |  |  |  |  |  |  |  |  |
| *D*max | 0.080 | **−0.860^***^** | −0.185 | **0.984^***^** |  |  |  |  |  |  |  |  |  |  |  |
| *K*t | −0.146 | −.526^*^ | 0.327 | **0.860***** | **0.858^***^** |  |  |  |  |  |  |  |  |  |  |
| SLA | −0.059 | −0.045 | 0.338 | 0.230 | 0.202 | 0.388 |  |  |  |  |  |  |  |  |  |
| LT | 0.169 | 0.220 | −0.341 | **−0.454^*^** | −0.382 | **−0.596**** | **−0.837***** |  |  |  |  |  |  |  |  |
| LD | −0.093 | −0.164 | −0.201 | 0.105 | 0.077 | −0.009 | **−0.790***** | 0.332 |  |  |  |  |  |  |  |
| C | −0.301 | 0.224 | −0.126 | −0.352 | −0.375 | −0.407 | **−0.676***** | **0.544*** | **0.585**** |  |  |  |  |  |  |
| N | 0.074 | −0.249 | 0.050 | 0.348 | 0.399 | 0.381 | 0.192 | −0.133 | −0.175 | −0.175 |  |  |  |  |  |
| C/N | −0.172 | 0.257 | −0.102 | −0.386 | **−0.442^*^** | **−0.443^*^** | −0.35 | 0.247 | 0.330 | **0.441*** | **−0.957***** |  |  |  |  |
| P | 0.251 | −0.132 | 0.111 | 0.203 | 0.159 | 0.249 | **0.592**** | **−0.549**** | −0.417 | **−0.617**** | 0.398 | **−0.530*** |  |  |  |
| N/P | −0.239 | 0.007 | −0.106 | −0.038 | 0.040 | −0.075 | **−0.536*** | **0.533*** | 0.343 | **0.572**** | 0.114 | 0.054 | **−0.865***** |  |  |
| K | 0.034 | −0.039 | **0.540^*^** | 0.357 | 0.355 | 0.619^**^ | **0.732***** | **−0.689***** | **−0.510*** | **−0.586**** | 0.228 | −0.375 | **0.602**** | **−0.539*** |  |
| δ13C | 0.041 | −0.291 | −0.236 | 0.257 | 0.294 | 0.145 | **−0.707^**^** | 0.432 | **0.744^**^** | 0.304 | 0.196 | −0.098 | −0.281 | 0.410 | −0.397 |

Significant correlations were in bold. See Table S1 for trait abbreviations. * *P* < 0.05; ** *P* < 0.01; *** *P* < 0.001.

**Table S4.** The effects of growth forms and leaf habits on trait variance with linear mixed-effects models.

| Dependent variables | Explaining variables | Sum of squares | *F* | *P* |
| --- | --- | --- | --- | --- |
| WD | GF | 0.021 | 6.3 | **0.005** |
|  | LH | 0.000 | 0.2 | 0.641 |
| VD | GF | 0.441 | 15.4 | **<0.001** |
|  | LH | 0.010 | 0.7 | 0.401 |
| VCA | GF | 0.015 | 1.0 | 0.378 |
|  | LH | 0.017 | 2.3 | 0.143 |
| Dh | GF | 0.293 | 43.6 | **<0.001** |
|  | LH | 0.019 | 5.8 | **0.022** |
| Dmax | GF | 0.333 | 57.4 | **<0.001** |
|  | LH | 0.020 | 7.0 | **0.012** |
| Kt | GF | 4.181 | 71.0 | **<0.001** |
|  | LH | 0.459 | 15.6 | **<0.001** |
| SLA | GF | 0.187 | 9.0 | **0.001** |
|  | LH | 0.329 | 31.6 | **<0.001** |
| LD | GF | 0.081 | 5.9 | **0.007** |
|  | LH | 0.021 | 3.1 | 0.090 |
| LT | GF | 0.025 | 2.7 | 0.084 |
|  | LH | 0.078 | 16.7 | **<0.001** |
| C | GF | 0.001 | 7.2 | **0.003** |
|  | LH | 0.001 | 13.9 | **0.001** |
| N | GF | 0.020 | 3.6 | **0.040** |
|  | LH | 0.014 | 5.0 | **0.033** |
| C/N | GF | 0.070 | 4.2 | **0.024** |
|  | LH | 0.064 | 7.8 | **0.009** |
| P | GF | 0.070 | 2.7 | 0.081 |
|  | LH | 0.129 | 10.2 | **0.003** |
| N/P | GF | 76.66 | 1.0 | 0.373 |
|  | LH | 2.570 | 0.1 | 0.795 |
| K | GF | 0.089 | 3.3 | **0.049** |
|  | LH | 0.152 | 11.4 | **0.002** |
| δ13C | GF | 0.012 | 1.2 | 0.317 |
|  | LH | 0.000 | 0.0 | 0.954 |

Significant effect was bold. See Table S1 for trait abbreviations.

**

**

**Figure S1.** Mean monthly rainfall (blue bars), mean monthly (grey circles), maximum (black circles) and minimum (white circles) air temperatures of the study site from 2014 to 2017. Climate data were provided by the Lijiang Forest Ecosystem Research Station, Kunming Institute of Botany, Chinese Academy of Sciences.





**Figure S2**. The phylogenetic tree of eight liana (red), eight scandent shrub (blue) and 21 tree (green) species.

**

**

**Figure S3.** Means (± SE) of leaf thickness (LT; a), nitrogen concentration (N; b), potassium concentration (K; c), C/N ratio (d), stable carbon isotope composition (δ^13^C; e) and vessel cross-sectional area (VCA; f) of eight liana (L), eight scandent shrub (SS) and 21 tree (T) species.
